# Impact of beverage temperature on consumer preferences for black coffee

**DOI:** 10.1038/s41598-022-23904-4

**Published:** 2022-11-30

**Authors:** William D. Ristenpart, Andrew R. Cotter, Jean-Xavier Guinard

**Affiliations:** 1 U.C. Davis Coffee Center, University of California Davis, 1 Shields Ave, Davis, CA 95616 USA; 2 Department of Chemical Engineering, University of California Davis, 1 Shields Ave, Davis, CA 95616 USA; 3 Department of Food Science and Technology, University of California Davis, 1 Shields Ave, Davis, CA 95616 USA

**Keywords:** Chemistry, Psychology and behaviour

## Abstract

We recently performed a systematic investigation of consumer preferences for black coffee versus key brewing parameters, including total dissolved solids, extraction yield, and brewing temperature (Cotter et al. in J Food Sci 86(1):194–205, 2021. 10.1111/1750-3841.15561). An experimental goal in that work was for participants to taste the coffee at a beverage temperature of 65 °C, but the large sample size of more than 3000 individual tastings, combined with natural variations in the brewing and cooling processes, meant that coffees were assessed over a normally distributed range of temperatures between 56 and 71 °C. Here we use those data to provide a more detailed analysis of the impact of beverage temperature on consumer acceptance of the coffee, with a key objective of identifying beverage temperatures at which no consumers assess the coffee either as too hot or too cold. Using a 5-point just-about-right (JAR) scale, we find that a majority of consumers (> 50%) assessed the temperature as JAR at all temperatures tested up to 70 °C. A substantial fraction of consumers, approximately 6–12%, assessed the coffee as too cold over the range 56–68 °C. Only above 70 °C did a majority of consumers assess the coffee as too hot and none assessed it as too cold, albeit with 40% still assessing it as JAR. Complementary analyses indicate that beverage temperature over this range had little impact on assessments of the adequacy of flavor intensity, acidity, and mouthfeel, but did correlate slightly with overall liking and purchase intent. Overall, the results suggest that temperatures over the range of 58–66 °C maximize consumer acceptance, and that 68–70 °C is the minimum temperature range at which no consumers will assess black coffee as too cold.

## Introduction

Because consumers expect their coffee to be hot, but not too hot, operators of coffeehouses, coffee shops, and cafés must make important decisions regarding the serving temperatures for their coffees. Clearly a key consideration is the preferred temperature range expected by consumers, but another important consideration is the risk of scald burns, which can cause tremendous injuries and occasionally result in high-profile litigation^1–3^. Accordingly, several groups have investigated consumer preferences for coffee versus serving temperature. Borchgrevink et al.^4^ examined coffee at seven distinct temperatures evenly spaced between 57.2 and 90.6 °C and collected data on adequacy of serving temperature on a 5-point just-about-right (JAR) scale. They found that of those tested a serving temperature of 68.3 °C yielded an average numerical score closest to JAR. Pipatsattayanuwong et al.^5^ examined coffee served at six distinct temperatures over a broader range from 39.2 to 82.1 °C, using pairwise R-index values derived from ranking data, and found that 72.1 °C was the most preferred temperature of those tested. Lee and O’Mahony used a different approach by letting consumers freely mix hot and cold coffee until they obtained a preferred temperature^6^. With this method, they found a much lower preferred temperature of 59.8 ± 8.1 °C, which they hypothesized was due to consumers adjusting the temperature to be suitable for drinking whole mouthfuls of coffee rather than smaller sips. Similar results were obtained more recently by Dirler et al.^7^, who also used a free-mixing method and found an average preferred temperature of 63 °C.

Notably, the above research focused on identifying the average preferred consumption temperature, and in each case found that consumers exhibited wide ranges of preferences. To date, however, no research has directly addressed an important question: at what minimum temperature do a negligible fraction of consumers assess the coffee as *too cold*? This question is important because of the intrinsic asymmetry that occurs in hot beverage consumption. Coffee rapidly cools in standard mugs or paper cups, so consumers who assess the initial temperature as *too hot* can simply wait a few minutes until a desired temperature is reached. In contrast, when a consumer assesses the initial temperature as *too cold*, there is little the consumer can do under typical conditions, and the coffee for these consumers will remain unsatisfactory as it becomes even colder. In this sense, the minimum temperature at which no consumers assess the coffee as *too cold* is arguably just as important a consumer metric as the average preferred temperature.

In this work, we present previously unanalyzed data from a large consumer preference study by Cotter et al.^8^. The analysis here has three objectives: (i) to document the effects of black coffee temperature on consumer acceptance of the coffee; (ii) to further our understanding of too cold, too hot and ideal coffee temperatures; and (iii) to help identify minimum and maximum temperatures acceptable to consumers. A total of 3186 ratings of the adequacy of coffee beverage temperature on a 5-point JAR scale were collected from 118 individual consumers for 27 coffee sample types tasted at temperatures ranging from 56 to 71 °C. In contrast to most prior published research on the effect of coffee beverage temperature, we also collected detailed information about consumer perceptions of flavor intensity, acidity, mouthfeel, overall liking, and purchase intent. The data presented here thus provide a more detailed view of how beverage temperature affects consumer preferences for black coffee.

## Methods

### Definitions

One must be careful when discussing “the temperature” of coffee, because the actual temperature of the beverage inevitably changes with time throughout the process of brewing, serving, and consuming. For the sake of clarity, we adopt the following terminology for temperatures:

- *Brew temperature* the temperature of the incoming hot water prior to contact with the coffee grounds in the brewer.
- *Post-brew temperature* the temperature of the brewed coffee in the carafe or urn immediately after completion of the brew cycle.
- *Holding temperature* the temperature of the brew while it is held in the carafe or urn prior to serving it; depending on whether the carafe is insulated or actively heated, the holding temperature can increase or decrease with time.
- *Post-pour temperature* the temperature of the brewed coffee immediately after it has been poured into a cup for a consumer; unless the cup has been pre-heated, this temperature is necessarily smaller than the holding temperature.
- *Tasting temperature* the temperature at which the consumer first tastes the coffee, potentially following a long delay after pouring.

Note the term “serving temperature” is frequently used but potentially confusing, since some people mean the holding temperature (measured in the carafe) while others mean the post-pour temperature (measured in the cup). Unless otherwise specified, here we use “serving temperature” to mean the post-pour temperature. We also use the term “beverage temperature” specifically to refer to the initial tasting temperature.

### Study overview

The data presented here are drawn from a large study that afforded an opportunity to assess consumer preferences for coffee tasting temperature^8^. Specifically, we used a 3 × 3 × 3 factorial design to test the hypotheses that brew strength, extraction yield, and brew temperature affect the sensory attributes and consumer preferences for drip-brewed black coffee. Three specific brew temperatures of 87, 90, and 93 °C were tested, with great care taken to adjust the grind size and flow rate such that the final brew strength and extraction yield were held constant despite the different brew temperatures. In brief, both descriptive analysis via an expert panel^9^ and consumer hedonic testing^8^ revealed that TDS had the largest effect, extraction yield had a secondary effect, and the original brew temperature had at most a weak impact. Here we analyze the large data set compiled by Cotter et al.^8^ to examine the impact of the actual tasting temperatures, rather than the initial brew temperatures, on consumer acceptance of the coffees. Specifically, our goal here is to test the hypothesis that consumer preferences for black coffee are correlated with tasting temperature, and that there exist temperature ranges at which no consumers will assess the beverage as *too hot* or *too cold.*

### Coffee service and temperature measurements

Full details regarding the type of coffee, water chemistry, and brewing protocols are available in^8^. In brief, a medium-roast, washed coffee from Honduras was used for all trials, using water with mineral content and pH recommended by the Specialty Coffee Association. Coffees were brewed using Curtis ThermoPro Single 1 Gallon Coffee Brewers, using one of three different set points for brew temperature (87, 90, or 93 °C), with the flow rate or grind size varied as necessary to achieve desired values for the TDS and extraction yield. The brewed coffee was immediately transferred into 1.0-L stainless steel insulated carafes for service to the consumers.

All tasting sessions were conducted in the Silverado Vineyards Sensory Theater at UC Davis, a room designed for food and beverage sensory testing. Dividers placed between consumers help maintain independence of evaluations. The number of consumers served in each session ranged from 12 to 26. Once a panelist was ready for their first/next sample, approximately 30 mL of coffee was poured from the appropriate carafe into a 120 mL paper hot cup (Solo Cup. Co., Highland Park, IL, USA). To allow the coffee to cool, as well as to minimize *tasting temperatures* differences resulting from differences in *brew temperature*, consumers were instructed, via a timer programmed into the survey, to wait 90 s after receiving the sample before taking their first sip and starting the evaluation. Consumers were provided with water and crackers to cleanse their palates between samples, and a cup to expectorate, if they wished to do so. Each consumer was served single-blind and in random order; they were provided no information about the temperature or any other aspect of the coffee.

For every tasting session, a research assistant sat in the Sensory Theater and was served coffees in exactly the same manner as the actual participants, but instead of tasting the coffees the research assistant measured the *post-pour temperature* and 90 s later measured the *tasting temperature*. In this fashion representative temperature data was obtained for all coffees as actually served.

A total of 118 consumers each tasted 27 coffees, yielding a total of 3186 individual tastings. The tastings were split over 18 separate sessions, meaning a total of 162 separate brews were prepared. The tasting temperatures had an overall mean of 64 °C, but with a wide range of 56–71 °C; the main goal of this paper is to assess any differences in how consumers liked and felt about the coffees within that range.

### Consumer test

This study was approved by the UC Davis Institutional Review Board (IRB# 1,082,568), and all research was performed in accordance with relevant guidelines and regulations. Participants gave informed consent, had freedom to withdraw at any time, and were compensated with a $25 gift card. We used RedJade (RedJade Sensory Software Solutions, Redwood City, CA, USA) to design and administer the questionnaire presented to the consumers. Each consumer filled out the questionnaire using their personal electronic device such as a smartphone, tablet, or laptop.

For each coffee sample, consumers first rated the adequacy of tasting temperature using a 5-point just-about-right (JAR) scale^10^. They then evaluated overall liking using the 9-point hedonic scale^11^. From there, they evaluated the adequacy of flavor intensity, acidity and mouthfeel using JAR scales. Then, they described the coffees by checking applicable descriptors from a check-all-that-apply (CATA) list. Finally, consumers indicated purchase intent ($3 for 12 oz cup) using a 5-point bipolar scale. All are described in^8^; here we focus on the JAR and 9-point hedonic data versus *tasting temperature*.

### Data and statistical analysis

All data analysis was performed using Python (Matplotlib version 3.5) and Microsoft Excel. The data are primarily in the form of JAR score distributions, so an important question is whether JAR scores at different tasting temperatures had statistically significant differences in their distribution. A key challenge with our experimental design is that the actual tasting temperature was not an independent variable specified a priori. Rather, the tasting temperature was a dependent variable that hinged on stochastic differences during the brewing process. The size of the consumer cohorts that assessed any particular tasting temperature therefore strongly varied with the tasting temperature itself; in other words, many consumers assessed coffees close to the mean tasting temperature, while fewer assessed coffees at the hotter and colder extremes of the tasting temperature range. This variation in cohort size, coupled with the lack of independence between assessors (since the same individuals tasted samples across the range of tasting temperatures), prevents statistical analysis using the customary $\chi^{2}$ method or Cochran–Mantel–Haenszel (CMH) method for JAR score distributions^12^.

Accordingly, we opted to follow two separate analysis procedures for our data. First, we binned all of the tasting temperatures into bins of 2 °C width, and prepared graphs denoting the JAR score distributions for coffees assessed at tasting temperatures within each bin. This representation provides the overarching trends for how tasting temperature affected consumer assessment across all 3186 assessments.

Second, to provide a more rigorous statistical assessment, we analyzed how each of the 118 consumers individually responded to maximally different tasting temperatures. Specifically, we compared three specific samples for each consumer: (i) the hottest coffee the consumer assessed, (ii) the coldest coffee the consumer assessed, and (iii) the coffee sample with a tasting temperature closest to the mean of all tasting temperatures the consumer assessed. For each of these three coffees, we compiled the JAR score distributions and calculated the mean and standard deviation of the actual tasting temperatures. Because all three coffee types (hottest, coldest, and closest to the mean) were assessed by the same 118 assessors, we are able to use CMH style methods for statistical testing. Here we used the Stuart-Maxwell method (a special form of the CMH method appropriate for a comparison between two products) to test the null hypothesis that the distributions are identical^13^ (cf. Appendix G in reference^12^). A type I error rate (α) of 0.05 was used as the threshold for reporting significant differences.

## Results

### Temperature distributions

**Figure 1:**
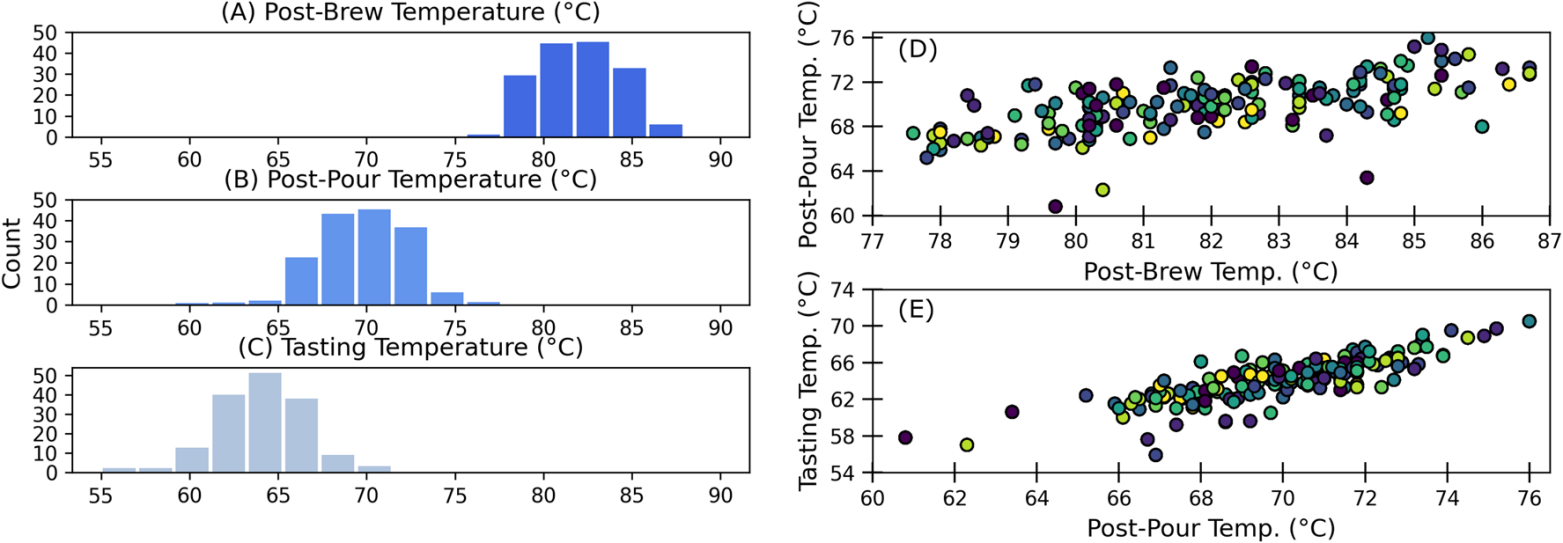
Temperature distributions for N = 162 separate brews. Histograms at left show the coffee temperature (**A**) immediately after completion of the brew, (**B**) immediately after pouring an approximately 30 mL sample into a paper cup, and (**C**) after a 90-s cooling delay and immediately prior to initial consumer tasting. Scatter plots in (**D**) and (**E**) show the correlation between post-brew temperature, post-pour temperature, and tasting temperature; different marker colors indicate different service order during a tasting session (with no apparent correlations).

Aggregate histograms of the post-brew temperature, post-pour temperature, and tasting temperature are shown in Fig. 1. Almost all the post-brew temperatures, as measured immediately after completion of the brewing cycle, varied between 79 to 85 °C with a handful of outliers (Fig. 1a). This observed 6 °C range is consistent with the purposefully imposed 6 °C range of brew temperatures (87, 90, or 93 °C), indicating that on average the liquid lost about 8 °C during contact with the initially room-temperature coffee grounds and thermal carafe. The post-pour temperature, as measured in the paper cup after exposure to the air and the initially room-temperature cup, decreased further on average by about 12 °C (Fig. 1b). The distribution of post-pour temperatures had a range similar in size to the post-brew temperatures, with most of the post-pour temperatures ranging from 66 to 74 °C. Finally, the temperature of most interest—the tasting temperature, measured after 90 s of cooling in the paper cup and immediately prior to sipping—had an absolute range of 56–71 °C, with a mean of 64.1 °C and a standard deviation of 2.4 °C. The vast majority of coffee samples were thus assessed between 59 and 69 °C (the mean plus or minus two standard deviations). Although the tails of this distribution are small, we emphasize that the large number of tasters meant that there were still many individual consumer assessments of the coffees at the extremes of the distribution. For example, the hottest and coldest of all 162 brews, served at 71 °C and 56 °C, respectively, were each assessed by 25 individuals.

Not surprisingly, the post-brew, post-pour, and tasting temperatures are highly correlated (Fig. 1d,e). The post-pour temperature is positively correlated with the post-brew temperature with a slope of 0.67 °C per °C, meaning every degree increase in the post-brew temperature yielded a post-pour temperature that was higher by 0.67 °C. Likewise, the tasting temperature was even more tightly correlated with a slope of 0.82 °C per °C, meaning that every degree increase in the post-pour temperature yielded a 0.82 °C increase in the actual tasting temperature. These correlations indicate that the cooling processes were similar across all 162 brews. The data instead suggest that variations in the actual tasting temperature primarily stemmed from variations in the post-brew temperature, as controlled by the imposed 6 °C range in brew temperature and the stochastic process of channeling during the brewing process.

### JAR ratings of adequacy of beverage temperature

**Figure 2:**
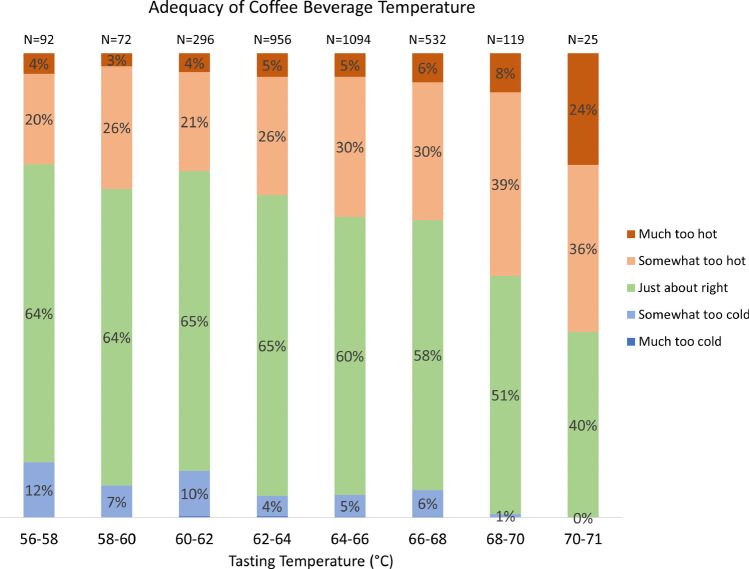
Adequacy of coffee beverage temperature on a 5-point JAR scale for a total N = 3186 individual tastings versus measured tasting temperature range (cf. Fig. 1). The number of individual consumer assessments performed at each temperature tasting range is denoted at the top of each column.

The impact of *tasting temperature* on consumer perception of the adequacy of beverage temperature is shown in Fig. 2. Each column represents a bin with width of 2 °C and is based on a different sample size of individual assessments, ranging from a minimum of N = 25 at the hottest tasting temperature of 71 °C, to a maximum of N = 1094 near the mean of 64 °C.

Several trends in the JAR scores are readily apparent. First, it is clear that a majority (> 50%) of consumer assessments found the beverage temperature to be just-about-right across almost the entire range of examined tasting temperatures (the green categories in Fig. 2). The proportion indicating just-about-right fluctuated around 65% at lower tasting temperatures, and then steadily decreased at tasting temperatures above 64 °C. Only at 71 °C did a majority of consumers assess the coffee as other than just-about-right (60% chose *too hot*).

The second main trend, not surprisingly, is that the proportion of consumers indicating *too hot* increased with tasting temperature (red categories in Fig. 2). At the lower temperatures, about 20–25% of the assessments were as *somewhat too hot*, and 3–5% were as *much too hot*. Above 64 °C, these proportions steadily increased. The proportion indicating *somewhat too hot* climbed from 30% to a peak of 39%, before diminishing slightly to 36% at the hottest temperature. Likewise, the proportion indicating *much too hot* increased from 6% to a peak of 24% at 71 °C.

The third and most surprising trend is the persistence of a substantial fraction of consumer assessments that specified *too cold* over a broad range of tasting temperatures (blue categories in Fig. 2). Although no consumers chose *much too cold* at any of the tasting temperatures examined here, many chose *somewhat too cold* over a wide range. At the coldest temperature of 56 °C, a full 12% indicated *somewhat too cold.* This proportion fluctuated a bit between 7 and 10% at temperatures up to 62 °C, and then decreased to about 4–6% at temperatures as high as 68 °C. Only at the range 68–70 °C did the proportion indicating *somewhat too cold* drop to 1%, before finally reaching 0% at 71 °C.

**Figure 3:**
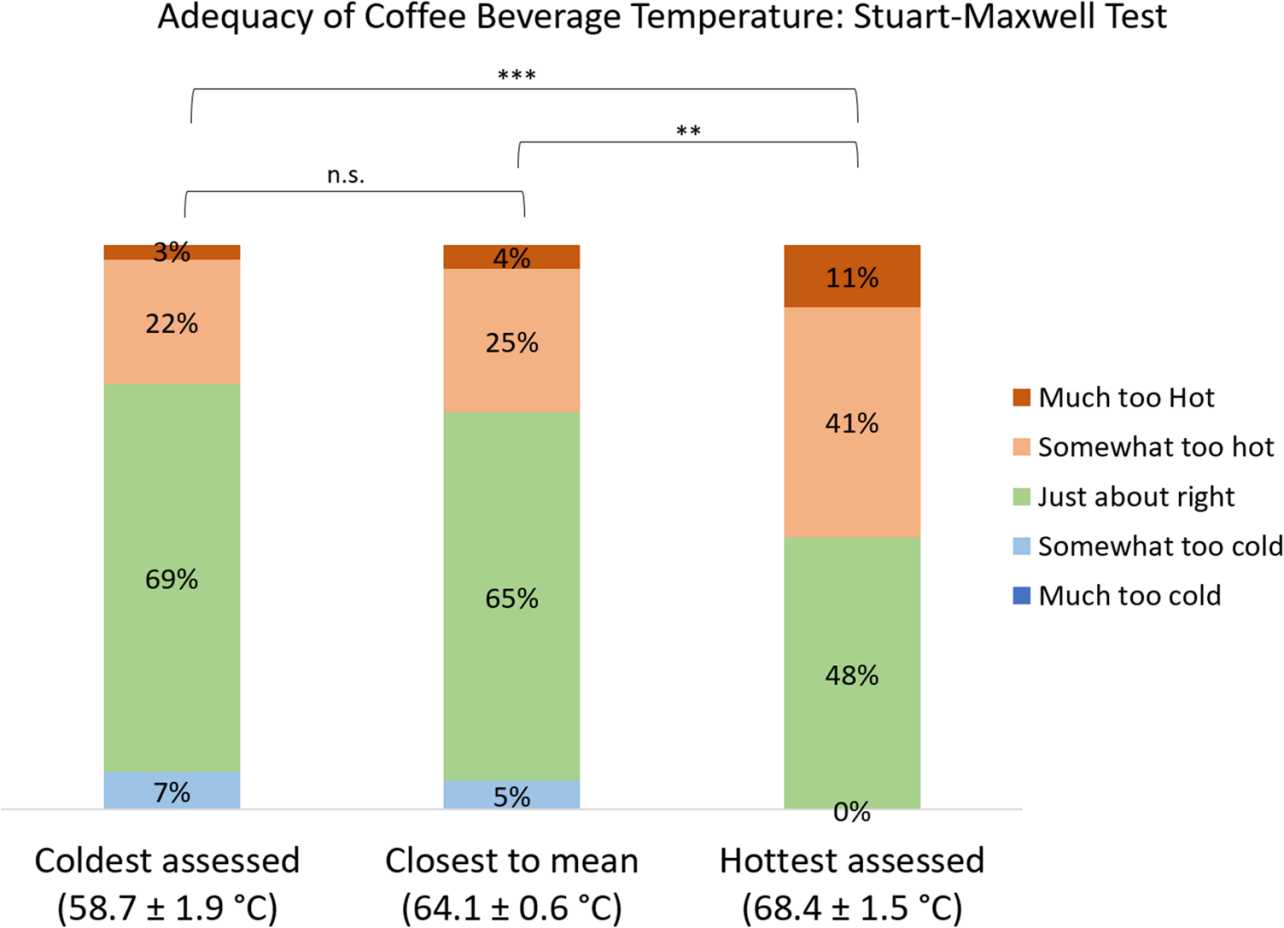
Adequacy of coffee beverage temperature on a 5-point JAR scale for three sample types: the coldest, the hottest, and the closest to the mean temperature of all coffees assessed by each individual consumer. Each column has the same sample size of N = 118 consumers. Statistically significant differences in JAR score distributions, as assessed using the Stuart-Maxwell method, are denoted with asterisks: *** for *p* < 0.001, ** for *p* < 0.01, or n.s. for not significant (cf. Table 1).

A similar strong dependence on tasting temperature is observed when we analyze the data in a different fashion more amenable to statistical analysis (Fig. 3). Here we show three JAR score distributions for adequacy of beverage temperature, where the three distributions represent how all 118 consumers responded individually to their specific hottest coffee, coldest coffee, and coffee closest to their mean tasting temperature. We emphasize that different consumers had different hottest, coldest, and mean tasting temperatures, since they tasted coffees on different days and different sessions that had naturally occurring differences in post-brew and tasting temperatures. Nonetheless, the variations in tasting temperature were small compared to the overall differences: the coldest coffees were tasted at 58.7 ± 1.9 °C, the closest to the mean coffees were tasted at 64.1 ± 0.6 °C, and the hottest coffees were tasted at 68.4 ± 1.5 °C, where the values represent the mean plus or minus one standard deviation.

The distributions in Fig. 3 make clear that there is a significant difference between the hottest assessed coffees and the colder coffees. None of the 118 consumers assessed the hottest coffee presented to them as *too cold*, but close to half (48%) indicated their hottest coffee was still *just about right*. The remainder indicated it was *somewhat too hot* (41%) or *much too hot* (11%). In comparison, the distributions for the coldest coffee and the coffee closest to the mean appreciably differ from the distribution for the hottest coffee. In both these colder distributions, substantial fractions of the consumers indicated the coffee was *somewhat too cold* (5–7%), about two thirds indicated it was *just about right*, and the remainder indicated it was *too hot* (25–29%).

Statistical significance testing using the Stuart-Maxwell method (Fig. 3 and Table 1) indicates we can reject the null hypothesis that the JAR score distributions are identical between the hottest tasting temperatures and the coldest; likewise, we can reject the null hypothesis that the JAR score distributions are identical between the hottest tasting temperatures and the tasting temperatures closest to the mean. In other words, the hottest coffees were statistically significantly different from the colder coffees. However, there is no statistically significant difference between the coldest coffees (near 58 °C) and those closest to the mean (near 64 °C). These findings accord with the qualitative trends shown in Fig. 2, where there is little variation at the lower temperatures tested and large changes at the hottest temperatures.

**Table 1:** Tabulation of ${\chi }^{2}$ and *p*-values calculated using the Stuart-Maxwell method, testing the null hypothesis that the JAR score distributions between the two samples are identical.

Sample Comparison		${\chi }^{2}$	$p$-value	Sig.
Hottest assessed (68.4 ± 1.5 °C)	Coldest assessed (58.7 ± 1.9 °C)	29.23	2 × 10^−6^	***
Hottest assessed (68.4 ± 1.5 °C)	Closest to mean temp. assessed (64.1 ± 0.6 °C)	15.91	0.00183	**
Coldest assessed (58.7 ± 1.9 °C)	Closest to mean temp. assessed (64.1 ± 0.6 °C)	1.40	0.705	n.s

The sample size is 118 assessors for each comparison, with three degrees of freedom. Statistical significance is denoted with asterisks as *** for *p* < 0.001, ** for *p* < 0.01, or n.s. for not significant. See Fig. 3 for the corresponding graphical representation of the JAR score distributions, and see supplementary Table S1 for the corresponding contingency tables.

### Overall liking, purchase intent, and sensory attributes

The preceding section focused on how the tasting temperature affected perceptions of the adequacy of the beverage temperature itself. Another key question is: how did the tasting temperature affect liking and perceptions of the beverage sensory attributes?

**Figure 4:**
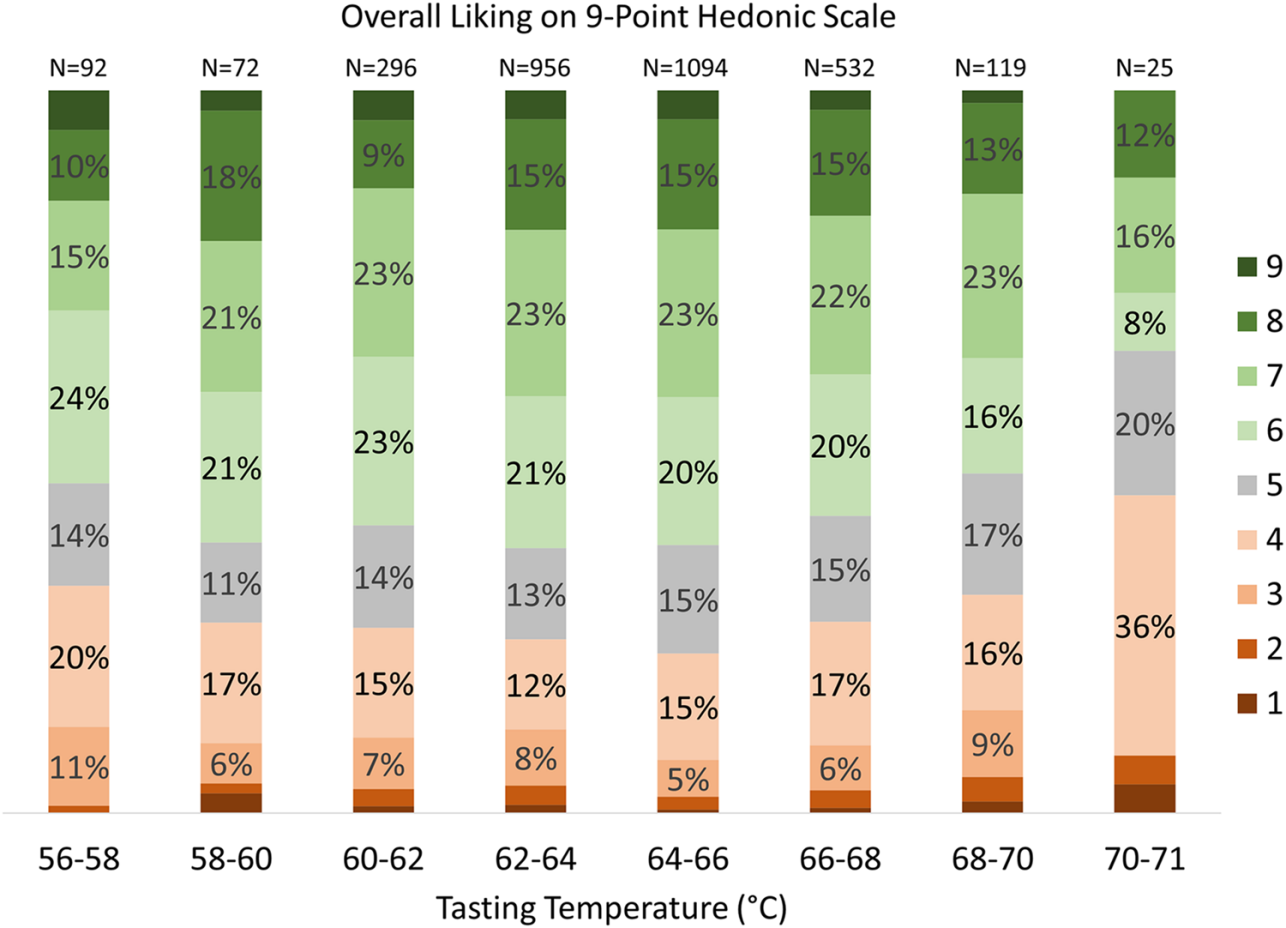
Overall liking of the coffee assessed with a 9-point hedonic scale versus measured tasting temperature range. The number of individual consumer assessments performed at each temperature tasting range is denoted at the top of each column (cf. Figure 2).

We first examined the overall liking of the coffee versus tasting temperature using a 9-point hedonic scale (Fig. 4). The data exhibit a slight increase in overall liking (green categories) as temperature increased from 56 toward 64 °C, followed by a gradual decrease in overall liking at even higher temperatures. The increase in dislike is most dramatic at the hottest temperature of 71 °C, with the overall proportion of dislikes (red categories) almost doubling.

**Figure 5:**
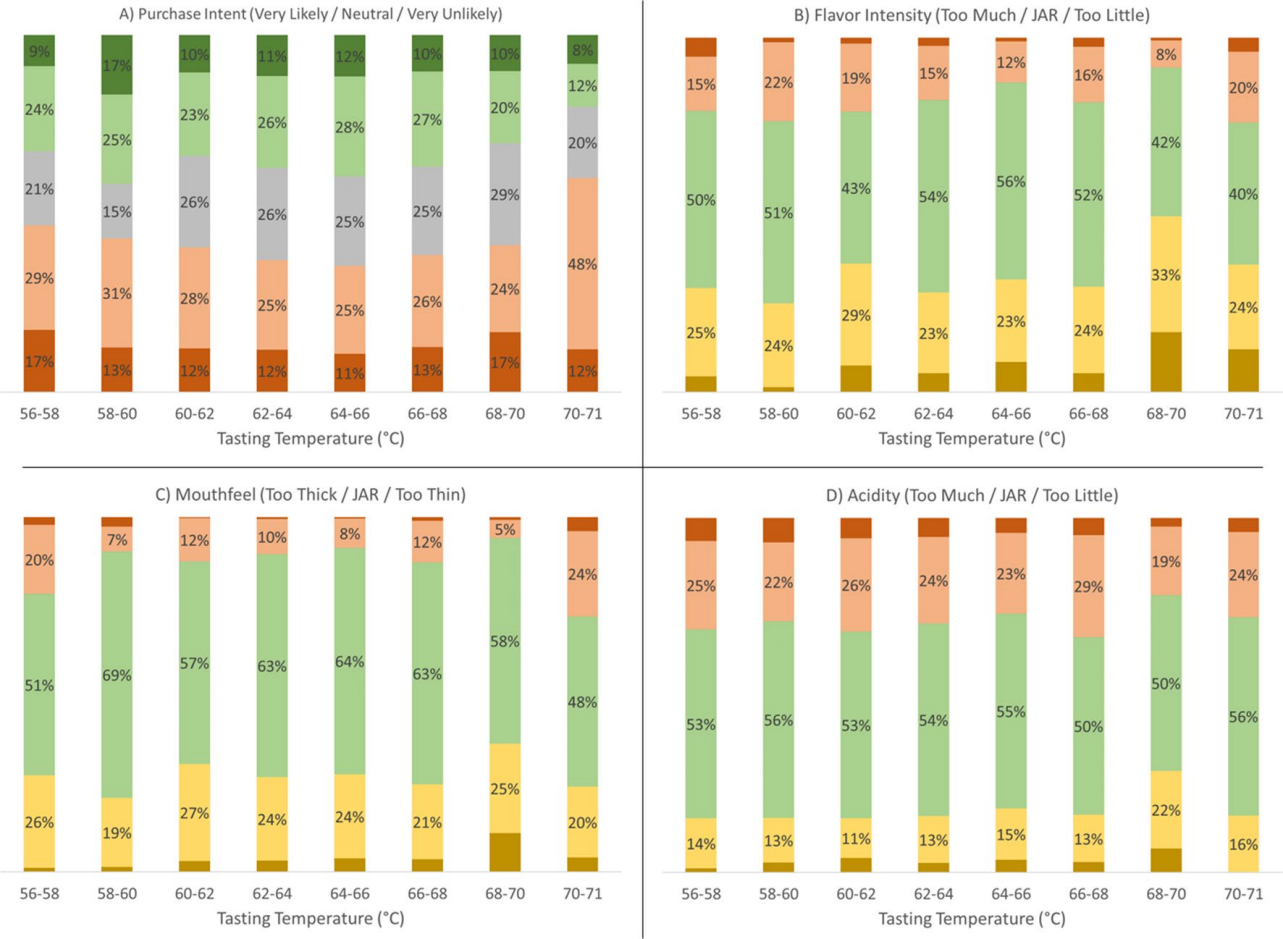
(**A**) Purchase intent at $3 for a 12-oz cup on a 5-point bipolar scale versus measured tasting temperature range. Green colors denote likely to purchase, gray denotes neutral, and red colors denote unlikely to purchase. (**B**, **C**, **D**) Adequacy of (**B**) flavor intensity, (**C**) mouthfeel, and (**D**) acidity on a 5-point JAR scale versus measured tasting temperature range. Red colors denote too much/too thick, green indicates JAR, and yellow colors indicate too little/too thin. In each chart, the number of individual consumer assessments at each temperature range is identical to those denoted in Figs. 2 and 4.

We also asked the consumers about purchase intent, at the $3 price point for a 12-oz cup of coffee (Fig. 5A). Over most of the range of tasting temperatures there is little variation, with roughly half of the assessments either *likely to purchase* or *neutral*. There appears to be a slight increase in purchase intent (green categories) as tasting temperature increased from 56 toward 64 °C, followed by a slight decrease in purchase intent at even higher temperatures. A larger decrease in purchase intent occurred at the hottest temperature of 71 °C, with the proportion shifting to a majority (60%) indicating they were unlikely to purchase it.

Figure 5 also shows the JAR score distributions for adequacy of (B) flavor intensity, (C) mouthfeel, and (D) acidity, using the same binning scheme and sample size distribution as in Fig. 2. The overarching trend is that the tasting temperature had little impact on perceived adequacy of these three sensory attributes. At all temperatures less than 68 °C, there are only very minor fluctuations in the observed JAR distributions, with the proportion indicating JAR for each attribute above 50% in almost all cases. Only at 68–70 °C is there an appreciable change in behavior, with the proportion of assessments indicating *too little flavor intensity, too thin,* and *too little acidity* all exhibiting an appreciable uptick. This uptick dissipates at 71 °C, however. The stark contrast with the smooth trends observed in Fig. 2 suggests that the uptick was a fluctuation due to an anomalously weak brew playing an outsize role in the small sample size at 68–70 °C. In any case, the key point is that there are no strong trends in these sensory attributes with respect to tasting temperature over the range tested.

**Figure 6:**
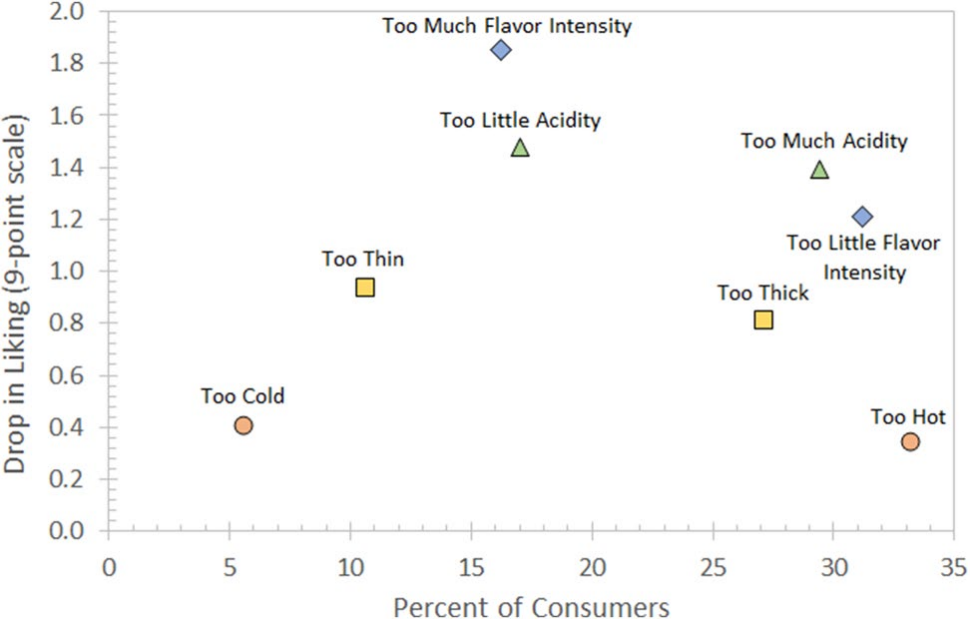
Penalty Analysis: drop in liking on the 9-point hedonic scale versus percentage of consumers selecting the ‘too low’ or ‘too high’ options for flavor intensity, acidity, mouthfeel (thick vs. thin) and tasting temperature.

Finally, we examined the relation between hedonic ratings and JAR ratings for key attributes in the coffees, including temperature. Figure 6 shows the penalty on the 9-point hedonic scale for the attribute being judged as ‘too low’ or ‘too high’, as well as the percentage of consumers who elected these JAR ratings. For example, and as stated earlier, a high percentage of consumers (33%) found the coffees to be too hot and a correspondingly small percentage of consumers (6%) found the coffees to be too cold. Similarly, more consumers found the coffees to be too thick, compared to too thin. Nonetheless, the main conclusion is that temperature had a much lesser impact on liking than other attributes did. Indeed, the temperature of the coffee being judged as too hot or too cold, regardless of how often that happened, only resulted in a penalty of about 0.4 on the 9-point hedonic scale. In contrast, flavor intensity and acidity, when inadequate (i.e., too low or too high), resulted in penalties of 1.2–1.9 on the 9-point hedonic scale. Likewise, the flavor intensity was deemed too low, and the acidity too high by a substantial percentage of consumers (about 30%).

## Discussion and conclusions

Taken together, the data presented here strongly suggest that a *tasting temperature* in the range of 58–66 °C will maximize consumer acceptance of the temperature of black coffee, with little variation in acceptance for coffees served in that range. Furthermore, the data indicate that *tasting temperatures* of 68–70 °C will ultimately maximize consumer acceptance of the temperature of black coffee by minimizing the number of consumers who perceive it as *too cold*. Only at that temperature range did 99% or more of tasters assess the beverage as *just about right* or *too hot*. At all temperatures tested below 68 °C a substantial fraction of consumers, ranging from 6 to 12%, assessed the beverage as *too cold*. Because consumers under typical conditions cannot heat up their coffee, but can readily wait a few minutes for the beverage to cool, a key conclusion is that initial *tasting temperatures* for black coffee should be at least 68–70 °C. Coffee service procedures that yield initial *tasting temperatures* less than 68 °C will leave sizable fractions of consumers dissatisfied with the temperature of their beverage.

The data also suggest that there is no benefit in terms of increased hedonic liking or purchase intent at higher *tasting temperatures*. To the contrary, both hedonic liking and purchase intent exhibited a slight but appreciable decrease at the highest tested temperature range (70–71 °C) compared to lower temperatures (albeit with the smallest sample size tested of N = 25). Presumably the increased perception of *too hot* at higher temperatures negatively impacted consumer acceptance of the beverage. The penalty analysis relating the hedonic ratings to the JAR ratings for flavor intensity, acidity, mouthfeel and tasting temperature suggest that temperature is not as important a factor in determining the liking for the coffee as flavor intensity or acidity might be. The penalty for the temperature being perceived as too cold or too hot penalized liking on the 9-point hedonic scale by less than 0.4 points, whereas it penalized by 1.2–1.9 points in the case of the flavor intensity or the acidity being inadequate (i.e., too low or too high).

A perhaps surprising result is that the perceived adequacy of flavor intensity, mouthfeel, and acidity all showed little difference with higher temperatures. Much research with calibrated expert panels has indicated that beverage temperature has a large impact on perceived sensory qualities^14–17^. These studies, however, examined much larger temperature variations than examined here.

We emphasize that the optimal temperature ranges identified here represent the *tasting temperature*, which necessarily means that the *brewing* and *holding temperatures* must be higher. The actual brew temperatures and holding temperatures necessary to achieve this optimal tasting temperature range depend greatly on the brewing process and serving volume. In our setup using commercial drip coffee brewers and vacuum insulated stainless steel carafes, we found that, on average, the post-brew temperature was 8 °C less than the brew temperature (i.e., the temperature of the hot water used to brew the coffee). Our post-pour temperature was 12 °C lower than the post-brew temperature, but this large decrease in temperature is partly due to the small volume poured into each cup (only 30 mL in this study). A smaller decrease in temperature is expected for a larger serving volume (because the mass ratio of hot beverage to cold cup is larger). Retail coffee operations should assess their actual *post-pour temperatures* for a range of brewing conditions and serving sizes to ensure their coffee is actually served at optimal temperatures.

An important aspect not considered here is that some consumers might desire their coffee to stay within their desired temperature range over a much longer waiting period (such as during a commute in a car). Since the coffee beverage will rapidly lose heat in standard to-go cups, the initial *post-pour temperature* must be increased to compensate. Calculation of the required increase, however, is not straightforward, since it depends on the ambient temperature, the serving size, and the thermal properties of the cup. Fortunately, however, our data suggest that consumer acceptance of the coffee temperature varies only slightly as the coffee cools from 68 to 56 °C. Although a sizeable fraction of consumers (6–12%) find the coffee *too cold*, about 58–65% of consumers found it *just about right* over this entire temperature range (meaning they could consume it immediately), with the remainder (24–36%) finding it *too hot* (and thus could simply wait for it to further cool). Consumer acceptance tests over a wider range of temperatures are necessary to assess more accurately when the coffee becomes unacceptably cold and to help inform more detailed calculations of ideal initial *post-pour temperatures* based on anticipated rates of cooling.

Finally, an important caveat is that our work only assessed black coffee. Many consumers, however, prefer to add sugar and cream (or other sweeteners and alternative milks)^18^. Addition of room-temperature sweeteners or chilled milk products will necessarily cool down the initially black coffee, meaning that the *holding temperature* might need to be increased further to compensate for these chilling effects. Because sweeteners and milk products will dramatically alter the flavor and mouthfeel characteristics of the coffee, it is likely that the preferred beverage temperature might also be altered. Further research is necessary to probe the ideal tasting temperatures for coffees with other additives.

## Supplementary Information

Supplementary Information.

## Data Availability

The datasets generated during and/or analyzed during the current study are available in the Dryad Digital Repository, https://doi.org/10.25338/B8993H.
