# Supplementary material for: Impact of beverage temperature on consumer preferences for black coffee
Source: Sci Rep. 2022 Nov 30;12:20621. doi: 10.1038/s41598-022-23904-4 (PMC9712614; doi:10.1038/s41598-022-23904-4)
Supplement: Supplementary file 1 — Supplementary Information. [file 41598_2022_23904_MOESM1_ESM.pdf]

Supplementary Material for
Impact of Beverage Temperature on Consumer Preferences for Black Coffee

William Ristenpart,[1,2] Andrew Cotter,[1,3] & Jean-Xavier Guinard[1,3]

[1] U.C. Davis Coffee Center, University of California Davis
[2] Dept. of Chemical Engineering, University of California Davis
[3] Dept. of Food Science & Technology, University of California Davis

Table S1 – Stuart-Maxwell contingency tables for the three statistical comparisons tabulated in Table 1 of the main text.

		Coldest assessed (58.7 ± 1.9 °C)				
		Somewhat too cold	Just about right	Somewhat too hot	Much too hot	*totals*
Hottest assessed (68.4 ± 1.5 °C)	Somewhat too cold	0	0	0	0	0
	Just about right	6	42	8	1	57
	Somewhat too hot	2	34	11	1	48
	Much too hot	0	5	7	1	13
	totals	8	81	26	3	118

		Closest to mean temperature assessed (64.1 ± 0.6 °C)				
		Somewhat too cold	Just about right	Somewhat too hot	Much too hot	*totals*
Hottest assessed (68.4 ± 1.5 °C)	Somewhat too cold	0	0	0	0	0
	Just about right	2	42	9	4	57
	Somewhat too hot	2	29	16	1	48
	Much too hot	2	6	5	0	13
	totals	6	77	30	5	118

		Closest to mean temperature assessed (64.1 ± 0.6 °C)				
		Somewhat too cold	Just about right	Somewhat too hot	Much too hot	*totals*
Coldest assessed (58.7 ± 1.9 °C)	Somewhat too cold	1	7	0	0	8
	Just about right	4	54	21	2	81
	Somewhat too hot	1	13	9	3	26
	Much too hot	0	3	0	0	3
	totals	6	77	30	5	118
